# A Longitudinal Mode Electromagnetic Acoustic Transducer (EMAT) Based on a Permanent Magnet Chain for Pipe Inspection

**DOI:** 10.3390/s16050740

**Published:** 2016-05-21

**Authors:** Ming Cong, Xinjun Wu, Chunqiao Qian

**Affiliations:** School of Mechanical Science & Engineering, Huazhong University of Science and Technology, Wuhan 430074, China; mingcong@mail.hust.edu.cn (M.C.); chunqiaoqian@mail.hust.edu.cn (C.Q.)

**Keywords:** longitudinal mode, pipe inspection, EMAT, permanent magnet chain, defect detection

## Abstract

A new electromagnetic acoustic transducer (EMAT) design, employing a special structure of the permanent magnet chain, is proposed to generate and receive longitudinal guided waves for pipe inspection based on the magnetostriction mechanism. Firstly, a quantitative analysis of the excitation forces shows the influence of the radial component can be ignored. Furthermore, as the axial component of the static magnetic field is dominant, a method of solenoid testing coils connected in series is adopted to increase the signal amplitude. Then, two EMAT configurations are developed to generate and receive the L(0,2) guided wave mode. The experimental results show the circumferential notch can be identified and located successfully. Finally, a detailed investigation of the performance of the proposed EMATs is given. Compared to the conventional EMAT configuration, the proposed configurations have the advantages of small volume, light weight, easy installation and portability, which is helpful to improve inspection efficiency.

## 1. Introduction

In recent years, the ultrasonic guided wave testing technique, with its advantages of long propagation distance, low attenuation and high testing efficiency, has been widely used in nondestructive testing (NDT) and structural health monitoring (SHM) [1,2,3]. The major approaches to generate ultrasonic guided waves are piezoelectric transducers [4,5], laser transducers [6] and electromagnetic acoustic transducers (EMATs) [7,8,9]. Among them, the EMAT can realize non-contact inspection based on electromagnetic coupling with the tested object. In general, an EMAT often consists of a permanent magnet to establish a static magnetic field and a testing coil to induce an eddy current or an alternating magnetic field. The selected guided wave mode can be easily generated in an EMAT by adjusting the shape and size of the permanent magnet and the testing coil. As one of the potential practical methods in field testing, the design and optimization of the EMAT configuration for different inspection requirements is of interest to researchers [10,11]. Because the generation and reception of the guided waves with EMATs are mainly based on the Lorentz force mechanism and the magnetostriction mechanism, different realized methods are studied.

For the magnetostriction mechanism, when the alternating magnetic field is exposed to a static magnetic field with a certain direction, the selected guided wave mode can be generated in ferromagnetic materials. Kwun *et al.* [12] proposed a simple configuration of a single-belt coil with a U-shaped permanent magnet to test the feasibility of generation and detection for longitudinal guided waves in pipes. To generate pure L(0,2) mode, Huang *et al.* [13] adopted a multi-belt coil for defect detection. Liu *et al.* [14] utilized three axisymmetric bias magnets to increase the amplitude of *L*(0,1) mode in seven-wire steel strands. Sun *et al.* [15] enhanced the excitation efficiency of the EMAT by using two copper rings to adjust the alternating magnetic field axial length. At the cost of the non-contact feature which leads to a low energy conversion efficiency of the EMAT, the team of Kim [16,17,18] proposed a series of magnetostrictive patch transducers (MPTs) by attaching a thin magnetostrictive patch on the tested object as a medium. Firstly a deformation is produced in the magnetostrictive patch and then delivered to the tested ferromagnetic or non-ferromagnetic material. As the magnetostrictive patch is made of a high magnetostriction coefficient material, an increasing energy conversion efficiency is achieved. Moreover, Liu *et al.* [19,20] adopted a multi-splitting meander coil and a modified planar solenoid array coil to generate longitudinal and torsional guided waves in pipes, respectively. Besides, the material, geometric shape and installation direction of the magnetostrictive patch were also studied to get a better SNR of the testing signal by the optimized MPTs [21,22].

On the other hand, for the Lorentz force mechanism, the Lorentz force can be produced by the interaction between the eddy current and the static magnetic field in both non-ferromagnetic and ferromagnetic materials. Thompson [23] developed a theoretical model to calculate the efficiency of the EMAT with a meander coil for Rayleigh waves and Lamb waves in plates. Wilcox *et al.* [24] demonstrated the feasibility of using a guided wave EMAT array with pancake coils to rapidly inspect large areas of steel plate structures. Seher *et al.* [25] proposed a low-frequency, omni-directional A0 Lamb wave EMAT on plates for applications in guided wave tomography by using the optimized parameters of the magnet diameter and the magnet lift-off. Thompson *et al.* [26] proposed a particular configuration called the periodic permanent magnet (PPM) to generate horizontally shear (SH) waves in an aluminum plate. Ogi *et al.* [27,28] used an improved PPM EMAT with an elongated-spiral coil to excite SH waves in pipes. Furthermore, a PPM EMAT array was adopted to analyze the conversion phenomenon of the torsional mode. In Ogi’s study, the PPM EMAT array was able to generate *T*(0,1) mode with a fixed wavelength, which was determined by the periodicity of the permanent magnet arrangement. However, considering the space in circumferential direction of the pipe was limited, an optimal element number of the PPM EMAT array was acquired to obtain the maximum Lorentz force [29]. As the elongated-spiral coils are configured under the PPM, the existence of the liftoff between the pipe and the PPM reduces energy conversion efficiency. In addition, although the torsional guided waves can be used to detect both circumferential and longitudinal defects, the sensitivity to circumferential defects is lower than the longitudinal guided waves, so for circumferential defects, the longitudinal guided waves are commonly utilized instead [30,31].

In this paper, a new EMAT design for defect detection in pipes is proposed to generate and receive longitudinal guided waves based on the magnetostriction mechanism. The radially polarized permanent magnets are uniformly configured around the pipe to form a chain structure. Firstly, a quantitative analysis of the excitation forces is discussed and a method of solenoid testing coils connected in series is adopted to increase the signal amplitude based on the axial component. Then, two EMAT configurations are developed to generate and receive L(0,2) mode. The experimental results show the circumferential notch can be identified and located successfully. Finally, a detailed investigation of the performance of the proposed EMATs is given.

## 2. Principle

Magnetostriction is a physical property of ferromagnetic materials that causes the shape or dimension change during the process of magnetization. Since elastic waves generated by the magnetostriction mechanism can be measured, significant efforts have been made on the development of the corresponding transducers to detect elastic waves.

### 2.1. The Principle of the Conventional EMAT

**Figure 1:**
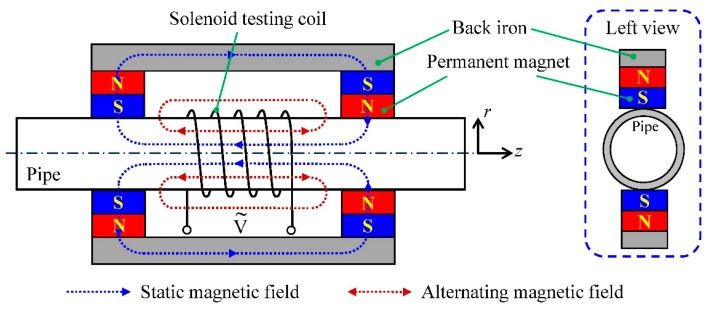
The conventional configuration of EMAT for longitudinal guided wave testing.

Figure 1 shows a conventional EMAT configuration for longitudinal guided wave testing of pipes. As shown in Figure 1, the conventional EMAT configuration consists of a back iron, permanent magnets and a solenoid testing coil. The back iron and permanent magnets connected together are often called the bias magnetizer. Considering the polarization directions of the permanent magnets arranged at two ends of the back iron are opposite, an axial static magnetic field is established along the pipe axis. The solenoid testing coil carrying an alternating current is wound around the pipe to supply an axial alternating magnetic field. As the static magnetic field is parallel to the alternating one, longitudinal guided waves can be generated and then propagate along the pipe in the axial direction.

For the magnetostriction mechanism, the strength of the static magnetic field in the tested object determines the magnetostrictive coupling coefficient, namely the energy conversion of EMAT from the alternating magnetic field induced by the testing coil to the elastic field transmitting in the tested object [32]. Therefore, a certain number of bias magnetizers could be uniformly configured around the pipe to meet the requirement of the static magnetic field strength. However, in the conventional EMAT, in order to obtain the pure axial magnetic field, two permanent magnets of the bias magnetizer need to be connected to establish a magnetic circuit return path by the back iron, which makes the EMAT large in volume and heavy in weight.

### 2.2. The Principle of the Proposed EMAT

**Figure 2:**
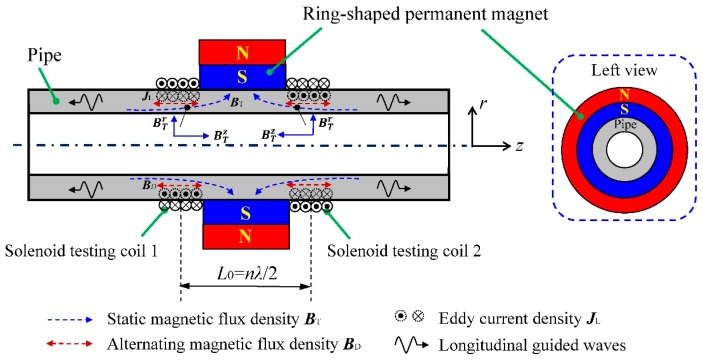
The configuration and testing principle of the proposed EMAT.

In order to overcome the shortcomings mentioned above, the relatively simple EMAT configuration shown in Figure 2 is proposed. The most remarkable feature is the absence of the back iron, which results in an obvious reduction of the volume and weight.

To guarantee the uniformity of the static magnetic field along the circumferential direction, a ring-shaped permanent magnet is used on the outer diameter surface of the tested pipe in this configuration. As the blue dotted lines show in Figure 2, the main direction of the static magnetic field changes from the axial to the radial when getting close to the ring-shaped permanent magnet along the pipe axis. Therefore, the static magnetic field ***B_T_*** consists of the radial component and the axial component near the permanent magnet, which can be expressed as:

(1) $$B_{T}=B_{T}^{r}e_{r}+B_{T}^{z}e_{z}$$

where $B_{T}^{r}$ and $B_{T}^{z}$ are the values of the radial and axial components of the static magnetic flux density, respectively, and ***e_r_*** and ***e_z_*** are unit vectors in the radial and axial direction, respectively.

Based on the magnetostriction mechanism, the solenoid testing coil should be arranged close to the ring-shaped permanent magnet to utilize the axial magnetic field component. Furthermore, in order to make full use of the axial component, two solenoid testing coils, separately arranged at the two sides of the permanent magnet, are connected in series to enhance the testing signal. The axial magnetostrictive force [32] under one belt of the solenoid testing coil can be described as:

(2) $$F_{ms}^{z}=-\frac{1}{2}\left(3\tau +2\mu \right)\left(1-2\upsilon \right)\frac{\partial \xi }{\partial M_{z}}\frac{\partial m_{z}}{\partial z}$$

where *τ* and *μ* are the Lame constants, *υ* is Poisson’s ratio, *ξ* is linear magnetostriction, ***M_z_*** is the axial static magnetic field magnetization intensity, ***m_z_*** is the axial alternating magnetic field magnetization intensity. According to Equation (2), the axial direction of the magnetostrictive force depends on the axial static magnetic field and the axial alternating magnetic field. To enhance the testing signal by waveform superposition, the relationship between the interval of the solenoid testing coils (*L*_0_) and the wavelength of longitudinal guided waves (*λ*) should satisfy:

(3) $$L_{0}=n\lambda /2$$

where *n* is a non-negative integer. The winding directions of these two solenoid testing coils should be in the opposite direction as *n* is even, while in the same direction as *n* is odd. Figure 2 shows an example of the case when *n* is even. Note that the wavelength of longitudinal guided waves is determined by the phase speed *C_p_* at the excitation frequency *f_e_*, Equation (3) can be further written as:

(4) $$L_{0}=nC_{p}/\left(2f_{e}\right)$$

which indicates the relationship between the interval of the solenoid testing coils and the excitation frequency.

On the other hand, based on the Lorentz force mechanism, the Lorentz force can be produced by the interaction of the static magnetic field and the circumferential eddy current. The circumferential eddy current density ***J_L_*** can be expressed as:

(5) $$J_{L}=j_{\theta }e_{\theta }$$

where *j_θ_* is the value of the circumferential eddy current density and ***e_θ_*** is unit vector in the circumferential direction. The Lorentz force can be calculated through:

(6) $$F_{L}=J_{L}\times B_{T}$$

Substituting Equations (1) and (5) into Equation (6), the Lorentz force is divided into two components as follows:

(7) $$F_{L}=F_{L}^{r}+F_{L}^{z}$$

(8) $$F_{L}^{r}=j_{\theta }B_{T}^{z}e_{r}$$

(9) $$F_{L}^{z}=j_{\theta }B_{T}^{r}e_{z}$$

where $F_{L}^{r}$ and $F_{L}^{z}$ are the radial and axial components of the Lorentz force loaded on the outer diameter surface of the tested pipe, respectively. Besides, it should be pointed out that the Lorentz force produced by the alternating magnetic field ***B_D_*** and the eddy current ***J_L_***, which is smaller than ***F_L_***, is ignored here [33].

For the proposed EMAT, except for the axial Lorentz force $F_{L}^{z}$, the radial magnetostrictive force $F_{ms}^{r}$ is also produced by the radial component $B_{T}^{r}$. As shown in Figure 2, the directions of the axial component $B_{T}^{z}$ are opposite at two sides of the ring-shaped permanent magnet while the directions of the radial component $B_{T}^{r}$ are the same. Based on the waveform superposition, the influence of the axial Lorentz force $F_{L}^{z}$ and radial magnetostrictive force $F_{ms}^{r}$ can be removed by the method of solenoid testing coils connected in series. Besides, as the magnetization force cancels the mentioned radial Lorentz force $F_{L}^{r}$ in the ferromagnetic material, the influence of the radial Lorentz force $F_{L}^{r}$ can be ignored [34].

According to above analysis, the longitudinal guided waves are mainly generated by the axial magnetostrictive force $F_{ms}^{z}$. The mentioned method can increase the amplitude of the testing signal as well as remove the influence of the radial component when Equation (3) is satisfied. In order to understand the influence of the radial component when Equation (3) is not satisfied, a quantitative analysis of the forces produced in the pipe is provided based on the simulation results in the next section.

## 3. The Development of the Proposed EMAT

According to the testing principle illustrated above, it seems that a radially polarized ring-shaped permanent magnet is an optimal choice for circumferential uniform magnetization, but on the one hand, a certain size ring-shaped permanent magnet can only be used to test the corresponding sized pipe due to the liftoff effect. On the other hand, it is impossible to install the ring-shaped permanent magnet on the pipe without open ends in the field. Therefore, a special structure needs to be developed to replace the ring-shaped permanent magnet. In this paper, the rectangle-shaped permanent magnets chain, which can encircle the pipe, is proposed to realize a rapid inspection for different sized pipes.

### 3.1. The Magnetization Unit

The permanent magnet, the element of the permanent magnet chain, appears periodically in the circumferential direction. As the axial magnetostrictive force is related to both the axial static and alternating magnetic field, the magnetization unit is a crucial component to determine the performance of the proposed EMAT.

**Figure 3:**
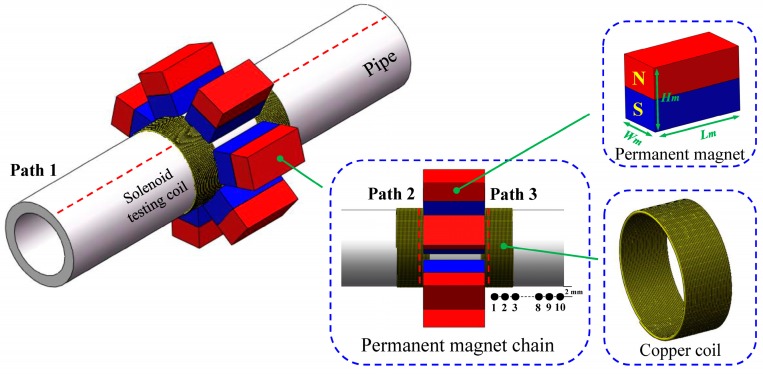
The simulation model of the proposed EMAT.

In order to give a quantitative analysis of the forces produced in the pipe, both the static and alternating magnetic field should be simulated using the electromagnetic field simulation module, ANSYS Emag. The simulation model contains the pipe, solenoid testing coils and the permanent magnet chain as shown in Figure 3.

A homogeneous isotropic steel pipe with outside diameter of 38 mm, wall thickness of 5 mm, is taken as an example. Seven rectangle-shaped permanent magnets, the length of 30 mm, the width of 15 mm and the height of 20 mm, are circumferential uniformly configured around the tested pipe with the same polarization direction. Two 20-turn solenoid coils wrapped by the copper wire are separately arranged at the edges of the permanent magnet chain. Some other parameters used in the simulation model are listed in Table 1.

For the pipe, the length and circumference are meshed with 1 mm and 2.5 degree, respectively. Due to the skin effect, the skin depth of eddy current is 35.6 μm at the frequency of 100 kHz. Therefore, a 100 μm region beneath the outer surface is meshed into 10 layers and the rest is meshed with 0.5 mm in the radial direction [29].

**Figure 4:**
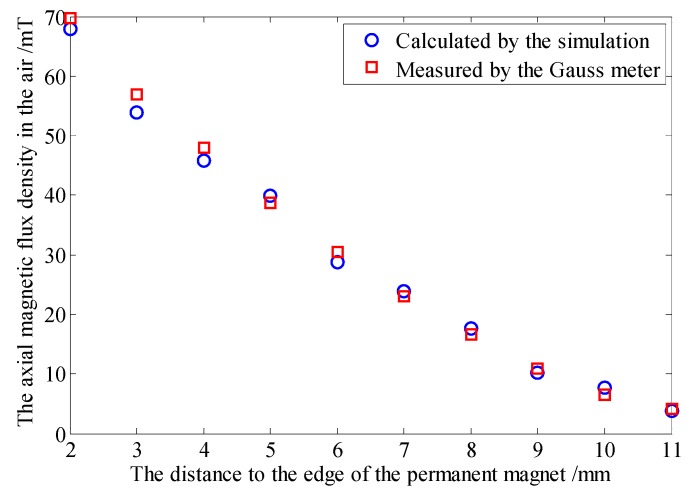
The comparison of the data obtained by the simulation and the Gauss meter.

A large block region, encircling the pipe, is set as air and meshed with the free grid. In order to investigate the precision of our simulation, a Gauss meter (BELL 8030, Pacific Scientific OECO, Milwaukie, OR, USA) is used to measure the axial magnetic flux density of the selected ten points which are 2 mm above the outer surface along the pipe as shown in Figure 3. The data obtained by the simulation and the Gauss meter are displayed in Figure 4. The maximum relative error is 4.79%, which indicates that the mesh is fine.

**Figure 5:**
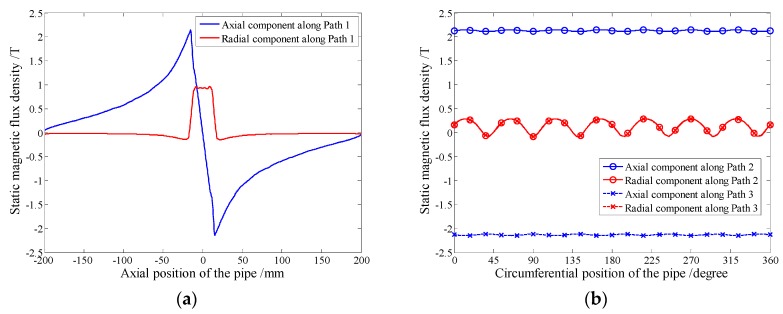
The static magnetic field distributions at 1 mm depth beneath the outer surface of the pipe along different paths (**a**) Path 1 (**b**) Path 2 and Path 3.

Three paths, 1 mm depth beneath the outer diameter surface of the pipe, are selected to analyze the static magnetic field. As Figure 3 shows, the one under the permanent magnet in the axial direction is defined as Path 1, the other two at the edges of the permanent magnet chain in the circumferential direction are defined as Path 2 and Path 3, respectively. The distribution of the static magnetic field along these three paths are shown in Figure 5.

Figure 5a shows the axial and radial component magnetic flux density along Path 1. The axial component has a peak value at the edges of the chain, where the solenoid testing coil should be arranged. Figure 5b shows the axial and radial component magnetic flux density along Path 2 and Path 3. Firstly, the radial components along Path 2 and Path 3 are almost overlapping, therefore, only three curves are displayed. Secondly, the maximal absolute value of the axial component magnetic flux density is 2.15 T and that of the radial component is only 0.25 T, which indicates the axial component is the dominant component. Thirdly, the directions of the axial component magnetic flux density are opposite along Path 2 and Path 3 while those of the radial component are the same. Therefore, the influence caused by the radial component can be removed.

**Figure 6:**
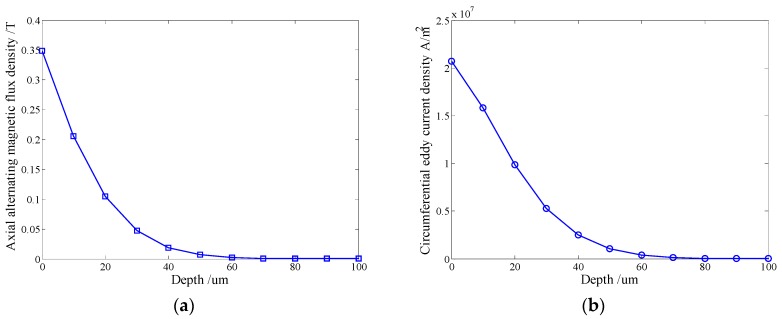
The distribution of (**a**) the axial alternating magnetic field (**b**) the circumferential eddy current density within 100 μm beneath the outer surface.

For the alternating magnetic field simulation, a five cycle sinusoidal current with the amplitude of 10 A at 100 kHz is loaded on the solenoid testing coil. The distribution of the axial alternating magnetic field and the circumferential eddy current under the solenoid testing coil are shown in Figure 6. The axial alternating magnetic flux density decreases rapidly within 100 μm beneath the outer surface of the pipe, so does the circumferential eddy current density.

Combing with the simulation results of the static field magnetic field, the forces under one belt of the solenoid testing coil can be calculated. According to Equation (9), the axial Lorentz force $F_{L}^{z}$ within the skin depth (35.6 μm) is about 0.023 N. And according to Equation (2), the axial magnetostrictive force $F_{ms}^{z}$ is about 0.732 N, which is approximately 32 times larger than the former one. On the other hand, the axial static magnetic field component $B_{T}^{z}$ is 6.14 times larger than the radial one. What’s more, the axial range of the axial static magnetic field component is much longer than that of the radial component as shown in Figure 5a.

Therefore, compared to axial magnetostrictive force $F_{ms}^{z}$, the radial magnetostrictive force $F_{ms}^{r}$ and the axial Lorentz force $F_{L}^{z}$ produced by the radial component can be ignored in the proposed EMAT, which will also be verified by experiments in the next section.

**Figure 7:**
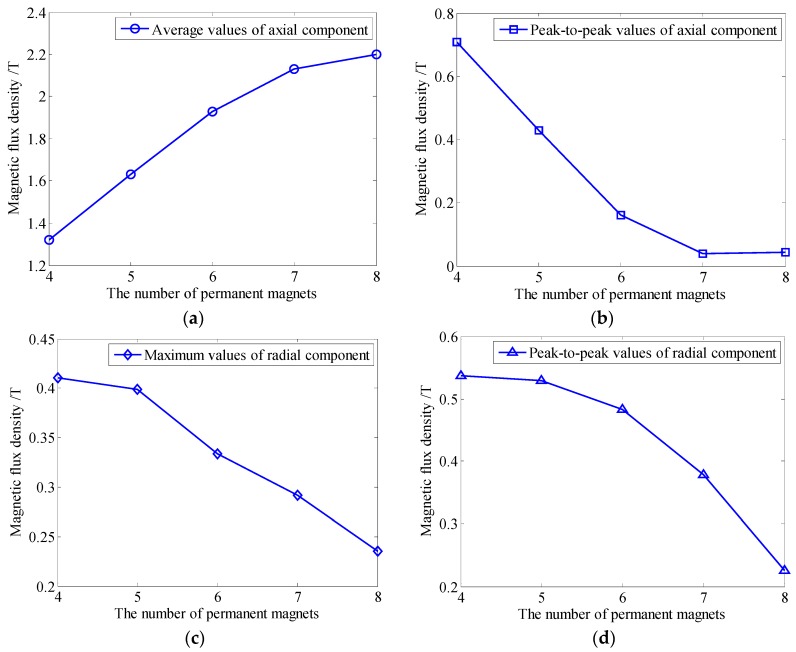
The variation of the static magnetic field with different numbers of permanent magnets along Path 2 (**a**) average values of axial component (**b**) peak-to-peak values of axial component (**c**) maximum values of radial component (**d**) peak-to-peak values of radial component.

It should be stressed that the permanent magnet chain is used instead of a ring-shaped permanent magnet for a widespread application, the circumferential uniformity of the static magnetic field should be considered. To achieve the uniform circumferential magnetization effect, the number of permanent magnets in the chain increases from four to eight. The variations of the static magnetic field along Path 2 is shown in Figure 7. As Figure 7a,b shows, the axial component of the static magnetic field firstly increases linearly with the number of permanent magnets and its increase becomes slow later when the number is 7. Meanwhile, its peak-to-peak values decrease linearly and remain unchanged when the number reaches 7, which means the circumferential uniformity of the axial component has achieved the optimum. While, the radial component decreases and becomes uniform in Figure 7c,d, resulting in less influence caused by the Lorentz force. According to the variation tendency of the axial component and the radial one, an increasing number of permanent magnets in the chain is beneficial to longitudinal guided wave generation. But the difficulty of the installation also increases due to the increasing interaction between adjacent permanent magnets in the circumferential direction. Therefore, the number of permanent magnets is set to 7 in the following study.

**Figure 8:**
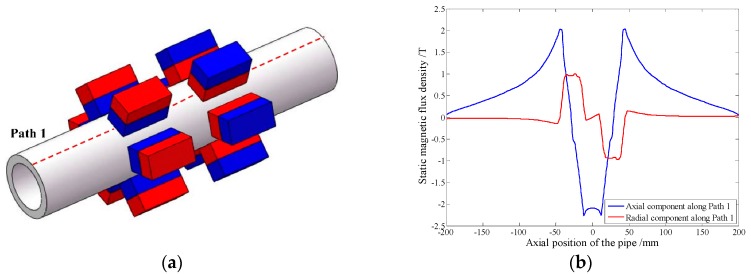
The EMAT configuration with two permanent magnet chains (**a**) the simulation model (**b**) the distribution of static magnetic field along Path 1.

As the bias magnetizer is utilized to supply an axial static magnetic field, another configuration is proposed without back iron by using two permanent magnet chains. In order to evaluate the ability of the magnetization, a corresponding simulation model is established with seven permanent magnets in each chain as shown in Figure 8a. The polarization directions of two chains are opposite and the distance between them is set to 25 mm. The distribution of the static magnetic field along Path 1, the same one used in the previous simulation model, is shown in Figure 8b.

Compared with Figure 5a, an extra axial component of the static magnetic field is established between two chains in Figure 8b, which is useful to generate longitudinal guided waves. Based on the theoretical analysis in Section 2, the method of three solenoid testing coils connected in series is adopted. Different from the bias magnetizer, the distance between these two permanent magnet chains can be adjusted. According to Equation (4), this EMAT configuration can obtain desired testing signals at different excitation frequencies.

**Figure 9:**
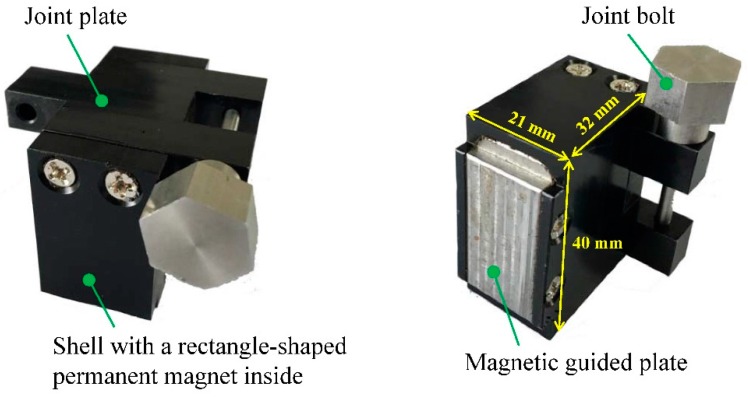
The photograph of the magnetization unit with different views.

Based on the distribution of the static magnetic field, a corresponding magnetization unit is designed to realize the inspection process. The photograph of the magnetization unit is displayed in Figure 9.

The magnetization unit consists of a shell with a rectangle-shaped permanent magnet inside, a joint plate, a joint bolt and a magnetic guided plate. The geometric parameters of the magnetization unit are shown in Figure 9. The permanent magnet is made of N52 with the same size used in the simulation, whose polarization direction is perpendicular to the pipe axis. The joint plate and joint bolt are interlinked together to connect with adjacent magnetization units. Both of them are made by aluminium alloy to avoid unnecessary magnetic energy loss. The magnetic guided plate, whose material is high permeability, is used to guide the magnetic field passing through and the shape is designed like a ‘boss’ so that the solenoid testing coil is allowed to arrange close to the permanent magnet.

### 3.2. The Chain Structure

**Figure 10:**
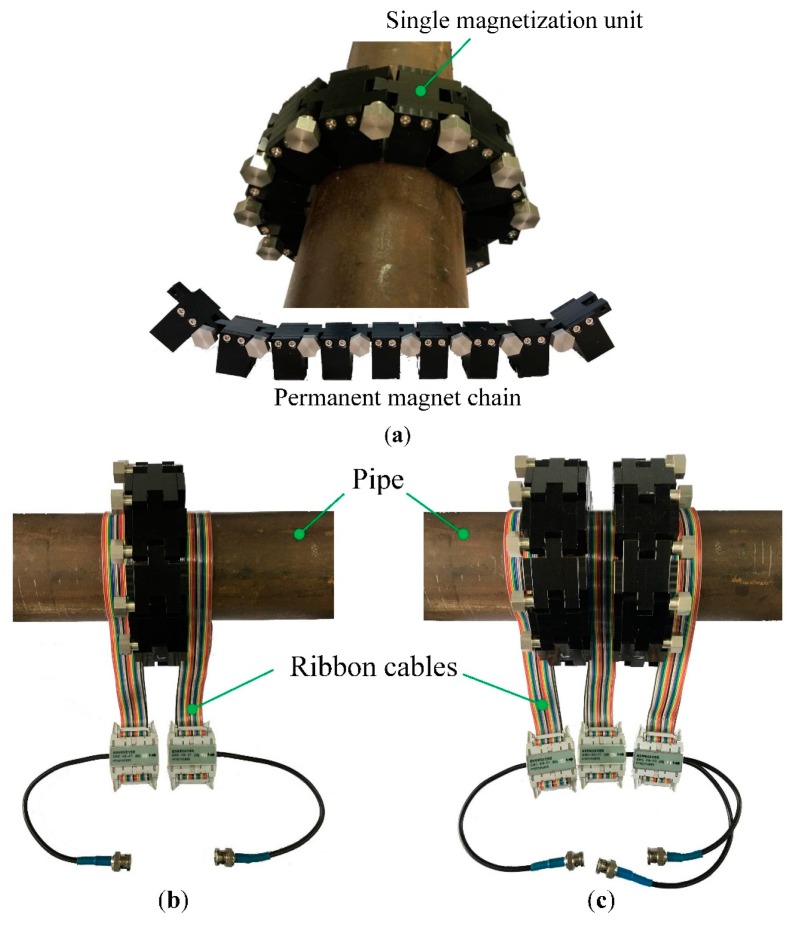
The photograph of the proposed EMATs (**a**) the permanent magnet chain (**b**) the configuration with one permanent magnet chain (**c**) the configuration with two permanent magnet chains.

As Figure 10a shows, the magnetization unit is connected with others to form the structure of the permanent magnet chain, and then it is installed on the tested pipe to supply a static magnetic field.

Compared with other magnetization apparatus, the chain structure has the advantages of small volume, light weight, easy installation and portability, which are helpful to improve inspection efficiency. It is suitable for the situation where the transducer installation space is limited. Moreover, the number of magnetization units can be adjusted for different sized pipes, resulting in a widespread application. Besides, for the convenience of inspection process, the ribbon cable carrying the alternating current is adopted to induce an axial alternating magnetic field. Both configurations of the proposed EMATs are illustrated in Figure 10b,c, respectively, and the ribbon cables can be conveniently connected. As for the EMAT configuration with two permanent magnet chains, the distance between these two permanent magnet chains can be easily adjusted.

## 4. Experimental Investigation of the Proposed EMATs

In order to test the feasibility of these two proposed EMATs, several experiments are carried out.

### 4.1. The Experimental Setup

In this experiment, a 3.2-meter-long pipe which material is steel 20 is used. The outside diameter of the pipe is 38 mm and wall thickness is 5 mm. The transmitter is placed at the left end of the tested pipe and the receiver is 850 mm away from the transmitter. Each of them contains one permanent magnet chain to provide a static magnetic field. Two 20-turn ribbon cables are connected in series to increase the amplitude of the testing signal. The axial width of each ribbon cable is 25 mm. Besides, in order to evaluate the ability of defect detection, a circumferential notch, the equivalent sectional area loss of 7.36%, is artificially made with a distance of 1.35 m away from the receiver.

**Figure 11:**
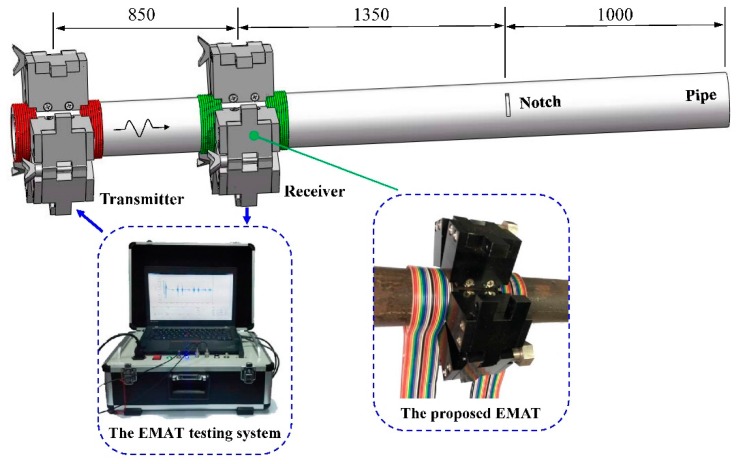
Schematic diagram of the experimental setup.

A self-developed EMAT testing system is utilized [35]. The experimental setup is schematically illustrated in Figure 11. In the experiment, a 5-cycle sine pulse, whose amplitude is 10 A, is applied to the coil. The induced voltage of the receiver coil is amplified with a gain of 70 dB. The bandwidth range is set from 5 kHz to 330 kHz and the sample frequency is 2 MHz.

**Figure 12:**
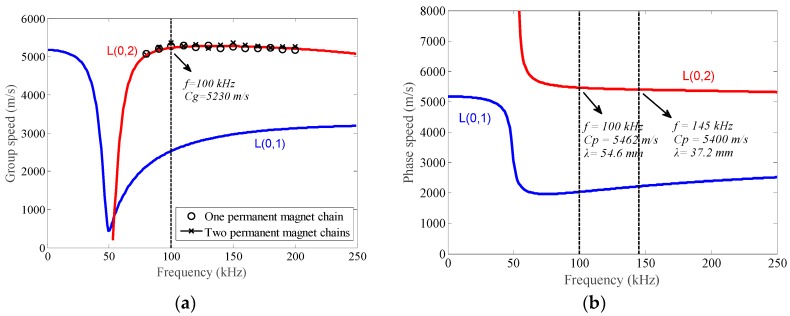
The dispersion curves of longitudinal modes for the tested pipe (steel 20) with the outside diameter of 38 mm and wall thickness of 5 mm through the Pcdisp (**a**) the group speed dispersion curve (**b**) the phase speed dispersion curve.

The free MATLAB code Pcdisp is utilized to compute the dispersion curves of the tested pipe [36]. Figure 12 shows the dispersion curves of longitudinal modes with a frequency range from 0 kHz to 250 kHz. To reduce the effect of dispersion during the propagation process, the excitation frequency within the range from 70 kHz to 200 kHz is chosen for L(0,2) mode. The interval of the ribbon cables is 55 mm, which is the distance between the centers of both ribbon cables. The point in the phase speed dispersion curve is 100 kHz with the wavelength of 54.6 mm, marked in Figure 10b. As the parameter *n* is 2, the ribbon cables in the transmitter and receiver should be connected in the opposite direction to improve the SNR of the testing signal, respectively.

On the other hand, the configuration of two permanent magnet chains with three ribbon cables is adopted as the transducers instead. To match the excitation frequency of 100 kHz, the interval of the adjacent ribbon cables is set at 55 mm and they also should be connected in the opposite direction.

### 4.2. The Defect Detection Performance

**Figure 13:**
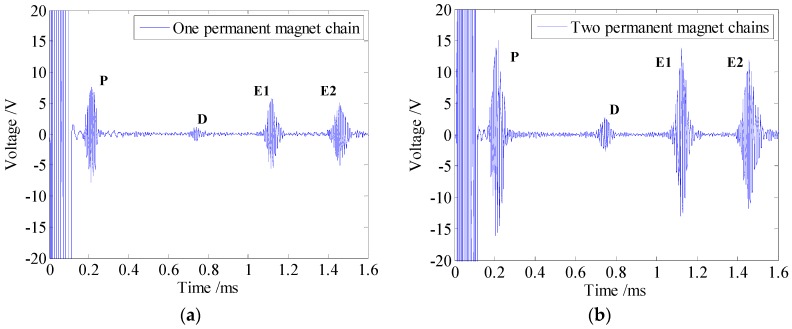
The testing signals received by the proposed EMATs with an average of 200 times (**a**) one permanent magnet chain (**b**) two permanent magnet chains.

According to the analysis above, a 5-cycle 100-kHz sine burst is chosen as the excitation signal. The testing signals received by the proposed EMATs with an average of 200 times are shown in Figure 13.

As shown in Figure 13, P is the passing signal which refers to the signal induced by guided waves propagating directly from the transmitter to the receiver. E_1_ and E_2_ signify the echo signals reflected from the right end and the left end of the tested pipe, respectively. An example is taken by the testing signal in Figure 13a. As the passing signal appears at 0.161 ms with the propagation distance of 0.85 m, the group speed is calculated to be 5279 m/s based on the time of flight (TOF) method. The relative error of the calculated group speed and the theoretical group speed is 0.94%, which means that the guided wave signal is L(0,2) mode and no other guided wave modes exist. Furthermore, a series of experiments are carried out with some other frequencies. The excitation frequency ranges from 80 kHz to 200 kHz with incremental steps of 10 kHz and the corresponding calculated group speeds are plotted with circles in Figure 12a. Besides, the results plotted with crosses are obtained through the similar experiments by using another EMAT configuration. The experimental results indicate that the calculated group speeds agree well with the theoretical ones, which further confirms the generated guided wave signal is L(0,2) mode for both EMAT configurations.

As we know, when guided waves encounter the defect or pipe ends, the corresponding reflected signals will generate. The small identifiable wave packet, existing between the passing signal (P) and right end-reflected signal (E_1_) at approximately 0.667 ms, is the notch-reflected signal, marked as D in Figure 13. The position of the notch is calculated to be 1.319 m from the receiver, which is close to the actual position of 1.35 m. Therefore, both proposed EMATs can effectively generate and receive L(0,2) mode, as well as identify and locate the notch in the pipe successfully.

In addition, as an extra axial magnetic field is established between two permanent magnet chains, the SNR of the testing signal obtained by this EMAT is 33 dB, 8 dB higher than the one obtained by the EMAT with one permanent magnet chain. As the amplitude of the noise remains the same, the SNR of the testing signal is improved remarkably, which is useful to detect the defect at a farther position with a smaller size. On the other hand, due to the fact that the static magnetic field between the permanent magnet chains will appear periodically along the pipe axis, it is not necessary to enhance the testing signal by keeping on increasing the number of the chains. Besides, wave packets in the testing signal will get elongated under the condition of more ribbon cables connected in series, which brings trouble for defect location.

**Figure 14:**
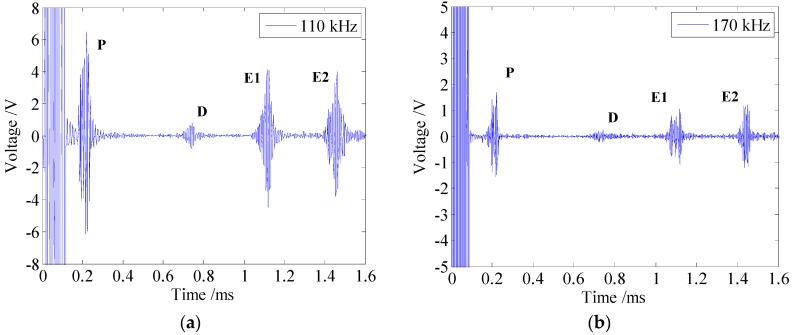
The testing signals received by the EMAT with one permanent magnet chain at the frequencies of (**a**) 110 kHz (**b**) 170 kHz.

In order to verify calculation results of the quantitative analysis in Section 3.1, the testing signals received by the EMAT with one permanent magnet chain at the frequencies of 110 kHz and 170 kHz are shown in Figure 14.

As Figure 14 shows, although the relationship between the interval of the solenoid testing coils (*L*_0_) and the wavelength of longitudinal guided waves (*λ*) no longer satisfies Equation (3), there are still no other modes existing in the testing signal, which is consistent with the calculation results.

### 4.3. The Frequency Response Features

When the interval of solenoid testing coils (*L*_0_) and the excitation frequency (*f*_e_) satisfy the certain relationship in Equation (4), the excitation frequency is called the optimal testing frequency of the EMAT in this paper. To investigate the frequency response features of the proposed EMATs, some experiments are carried out with different excitation frequencies which range from 70 kHz to 170 kHz with incremental steps of 5 kHz. The experimental setup is the same as Figure 11, except that the EMAT configurations of one permanent magnet chain and two permanent magnet chains are used as the receivers, respectively. Peak-to-peak values of the passing signal at different frequencies are measured and normalized with respect to the maximum value.

**Figure 15:**
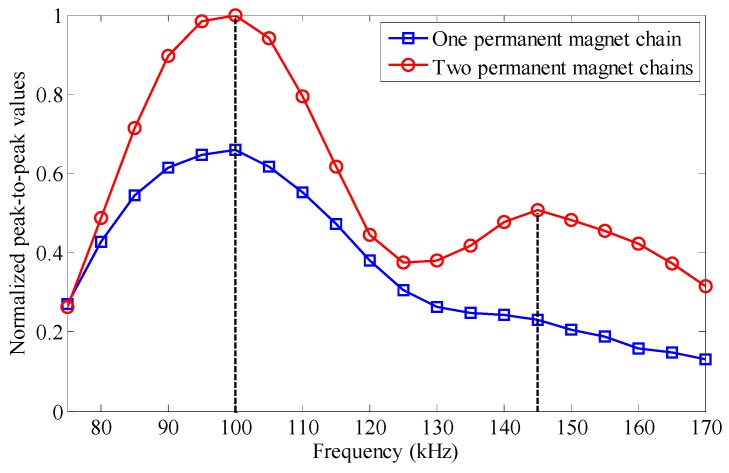
The frequency response features of the proposed EMATs.

The maximum value appears at the frequency of 100 kHz on both curves in Figure 15, which is consistent with the theoretical result. The peak-to-peak values obtained by two permanent magnet chains (the red curve) are higher than those obtained by one permanent magnet chain (the blue curve) throughout the entire frequency range, which indicates that the performance of the EMAT is successfully improved with multiple chains. However, the red curve is steeper near the maximum value than the blue one, so a more exact excitation frequency selection is required when using the EMAT with two permanent magnet chains.

Moreover, for the EMAT with one permanent magnet chain, the peak-to-peak values decrease gradually as the excitation frequency deviates from the optimal testing frequency, resulting in only one maximum value in the curve. But for the EMAT with two permanent magnet chains, there exists another peak at the frequency of 145 kHz. According to the phase speed dispersion curve shown in Figure 12b, the wavelength of L(0,2) mode at 145 kHz is about 37.2 mm. The interval of the ribbon cables at two edges of the EMAT is 110 mm, which is just three times the wavelength of L(0,2) mode at 145 kHz. Note that the axial components of the static magnetic field are in the same direction, the connection method of these two ribbon cables still satisfies Equation (4), namely *n* = 3. That’s the reason why the red curve has another peak at 145 kHz and it makes the EMAT with two permanent magnet chains have a large frequency range for pipe inspection.

### 4.4. The Adjustable Optimal Testing Frequency of the Proposed EMAT

**Figure 16:**
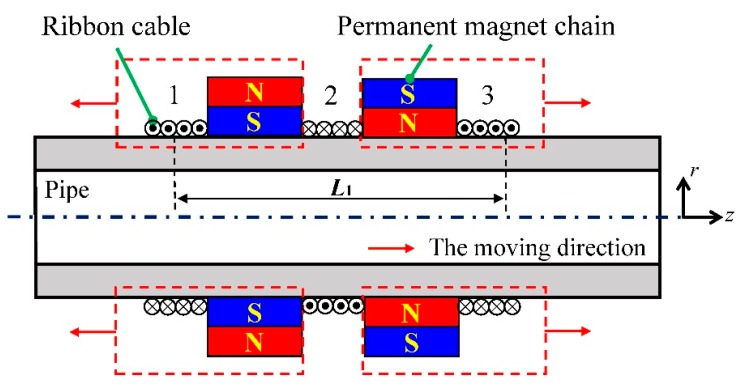
Schematic diagram of the method for the interval adjustment.

As the dispersion curve is related to the material parameters and geometric parameters of the tested pipe, the suitable excitation frequency for defect detection may change in different conditions. So the configuration of the transducer should be available to change the optimal testing frequency during the inspection process. For the EMAT with two permanent magnet chains, moving the ribbon cable together with the permanent magnet chain along the pipe is a simple method to achieve this goal as shown in Figure 16.

The experiment setup is the same as the one used in Section 4.3, the excitation frequency ranges from 100 kHz to 80 kHz with the decrescent step of 5 kHz. The intervals of ribbon cable 1 and 3 (*L*_1_) adjusted to the values listed in Table 2, which are twice the wavelength of L(0,2) mode at each frequency.

**Figure 17:**
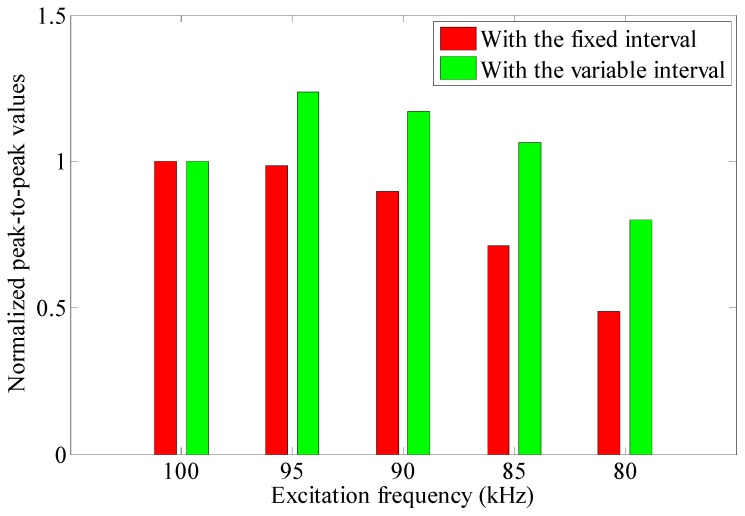
The experimental results by adjusting the optimal testing frequency.

The initial interval is 110 mm and the peak-to-peak values of passing signal obtained with this fixed interval at each excitation frequency are used as baseline data. Then, adjust the interval to the values listed in Table 2 one by one and record the same parameter as a comparison. All of the data are normalized with the value obtained with the initial interval for each case. The experimental results are illustrated in Figure 17.

Compared with the EMAT which does not work at its optimal testing frequency, an obvious enhancement of the testing signal is achieved when the interval *L*_1_ matches the wavelength of L(0,2) mode at different frequencies. As the interval between the permanent magnet chains can be adjusted, the EMAT realize the signal enhancement by changing the optimal testing frequency. However, the axial component of the static magnetic field between two permanent magnet chains decreases with the increase of the interval, the effect of signal enhancement gradually reduces.

## 5. Conclusions

In this paper, a new electromagnetic acoustic transducer (EMAT) design based on a permanent magnet chain is proposed to generate and receive longitudinal guided waves for pipe inspection. The permanent magnets are uniformly configured around the tested pipe to form a chain structure. According to the results of the quantitative analysis, the influence caused by the radial component can be ignored in the proposed EMAT. Furthermore, a method of solenoid testing coils connected in series is utilized to increase the amplitude of the testing signal.

Two corresponding EMAT configurations are developed to realize the inspection process by connecting magnetization units to form a permanent magnet chain. Compared with the conventional configuration, it has the advantages of small volume, light weight, easy installation and portability, which are helpful to improve inspection efficiency. The proposed EMATs can generate and receive L(0,2) mode. The experimental results indicate that the circumferential notch can be identified and located in the pipe successfully. Moreover, the configuration of two permanent magnet chains is able to enhance the testing signal but requires a more exact excitation frequency selection when compared with the configuration of a permanent magnet chain. The optimal testing frequency of the configuration of two permanent magnet chains can be adjusted to meet different inspection requirements.

However, the proposed EMATs are still in the early stage of development and more work needs to be done. A small sized pipe is discussed, but the performance of the EMATs for thick-walled pipe needs further research. Besides, the performance in a field test also needs to be investigated. Considering the results of the laboratory-based experiment as well as the advantages of easy installation and portability, the proposed EMATs have potential for NDT and SHM.

## Figures and Tables

**Table 1:** Parameters of the simulation model used in this paper.

Object	Parameters	Symbol	Value
**Pipe**	Outer diameter	*D_p_*	38 mm
	Wall thickness	*T_p_*	5 mm
	Length	*L_p_*	400 mm
	Density	ρ	7850 kg/m^3^
	Young’s modulus	*E*	210 GPa
	Poisson’s ratio	*v*	0.28
	Resistivity	ρ*_p_*	1.4 × 10^−7^ Ω·m
	Relative magnetic permeability	*μ_r_*	200
**Air**	Length	*L_a_*	800 mm
	Width	*W_a_*	150 mm
	Height	*H_a_*	150 mm
	Relative magnetic permeability	*μ_a_*	1
**Permanent magnet**	Length	*L_m_*	30 mm
	Width	*W_m_*	15 mm
	Height	*H_m_*	20 mm
	Number	*N_m_*	4 to 8
	Coercive force	*H_m_*	955 kA/m
	Residual magnetic flux density	*B_r_*	1.45 T
**Solenoid testing coil (Copper coil)**	Diameter	*D_c_*	0.17 mm
	Turn number	*N_c_*	20
	Lift-off distance	*L_c_*	0.54 mm
	Interval	*I_e_*	1.25 mm
	Resistivity	ρ*_c_*	1.7 × 10^−8^ Ω·m
**Excitation current**	Amplitude	*I_e_*	10 A
	Frequency	*f_e_*	100 kHz
	Cycle	*T_e_*	5

**Table 2:** The interval *L_1_* for each frequency in the experiment.

**Frequency/kHz**	100	95	90	85	80
***L*_1_/mm**	109.2	115.2	122.0	129.8	138.6
